# Do we get better outcomes from early treatment of Class III discrepancies?

**DOI:** 10.1038/s41415-022-4507-0

**Published:** 2022-08-12

**Authors:** Andrew T. DiBiase, Jadbinder Seehra, Spyridon N. Papageorgiou, Martyn T. Cobourne

**Affiliations:** 41415134835001 Department of Orthodontics, East Kent Hospitals University NHS Foundation Trust, William Harvey Hospital, Ashford, UK; 41415134835002 Centre for Craniofacial & Regenerative Biology, Faculty of Dental, Oral & Craniofacial Sciences, King´s College London, London, UK; 41415134835003 Clinic of Orthodontics and Paediatric Dentistry, Centre of Dental Medicine, University of Zurich, Zurich, Switzerland

## Abstract

Early orthodontic treatment in the mixed dentition aims to simplify definitive treatment in the permanent dentition. In Class III cases, this can be an effective strategy for the management of a local anterior crossbite, using either a removable or simple fixed appliance. For more significant Class III malocclusions, the decision to intervene early is a more difficult one. Traditionally, orthodontists in the UK have been reluctant to embark on early treatment in the presence of a skeletal Class III relationship but there is now some evidence that in selected cases, the use of protraction headgear can be a successful method of avoiding the need for later surgery. Although growth prediction in Class III cases is notoriously difficult, in the presence of maxillary retrognathia, the general dental practitioner should consider early referral of Class III cases to a specialist orthodontist.

## Key points

- Early correction of a local incisor crossbite should be considered in the presence of any occlusal dysfunction and most young children will cope easily with the required treatment.
- For those Class III discrepancies associated with maxillary retrusion, early treatment with protraction headgear and maxillary expansion can reduce the need for orthognathic surgery in the late teenage years and early referral to a specialist is advised.
- Prediction of success is difficult although the presence of increased vertical proportions and a reduced overbite are indicators that early intervention might be less successful over the longer term.

## Introduction

There are few areas of orthodontics that generate quite so much debate as the concept of early treatment. This normally refers to active intervention by an orthodontist during the mixed dentition to correct an underlying malocclusion with the aim of simplifying definitive treatment in the permanent dentition and ultimately, improving final outcomes for the child. However, this strategy can be associated with increased overall treatment length, involving more appointments, and increased cost, while the benefits within the context of long-term care for children with malocclusion are unclear. In recent years, useful data have become available in relation to the early treatment of different orthodontic problems and here we explore the current evidence on management of Class III malocclusion.

## Early correction of an anterior crossbite

**Fig. 1:**
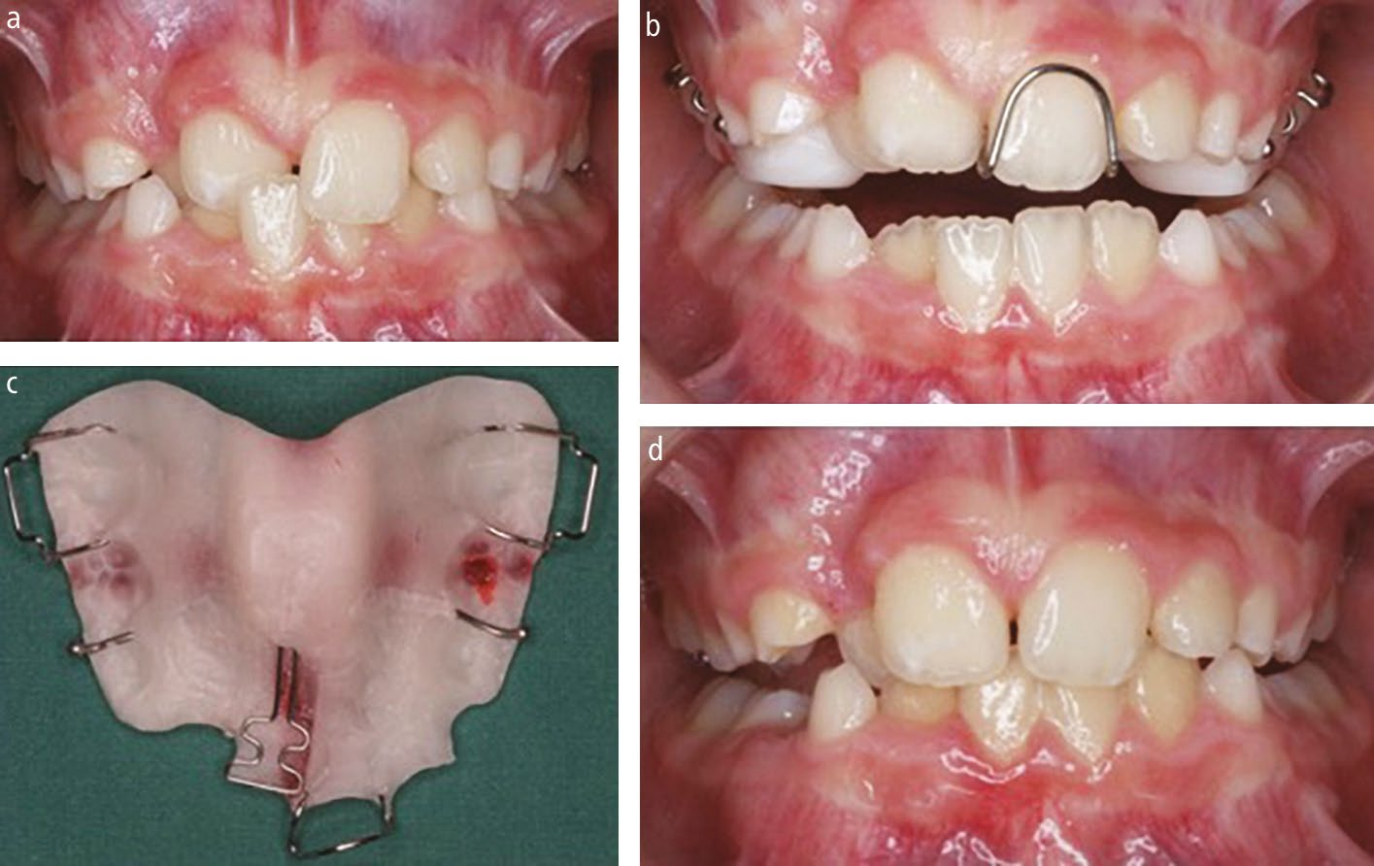
a, b, c, d) Early management of a localised anterior crossbite using an upper removable appliance

**Fig. 2:**
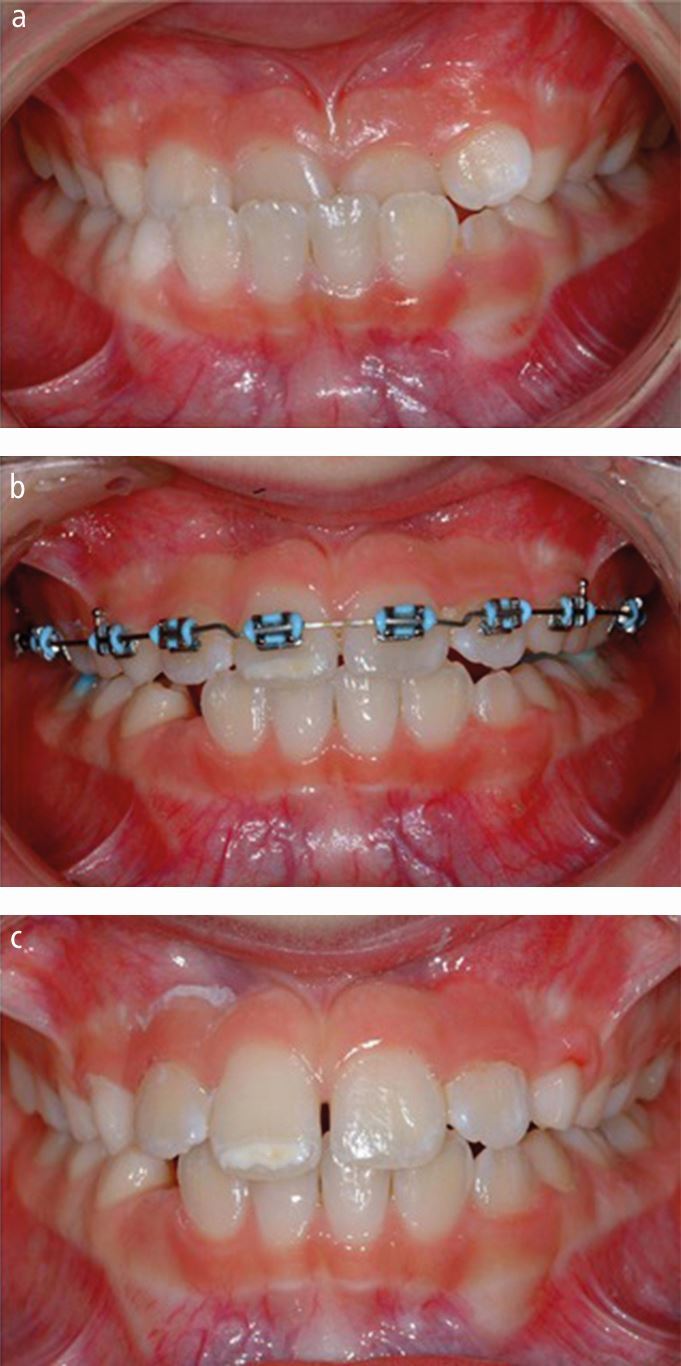
a, b, c) Early management of a localised anterior crossbite using sectional upper fixed appliance

The development of a Class III malocclusion in the early mixed dentition is a common reason for a parent to seek an opinion from their general dental practitioner. The so-called Pseudo-Class III malocclusion with one or more lingually tipped maxillary incisor teeth and an anterior crossbite associated with a displacement or functional shift of the mandible has long been regarded as a condition that potentially benefits from early correction.^1^ The rationale suggests that the continued presence of such a relationship can be detrimental to long-term occlusal health; it can be associated with local displacement and recession of the incisor teeth,^2^ encourage the development of a true Class III malocclusion, precipitate adverse growth and development of the jaws and potentially increase the risk of temporomandibular dysfunction.^3^ Although the absolute benefits of early occlusal correction are not clear, particularly in relation to occlusal and temporomandibular health, there is high-quality evidence that treatment of a local crossbite can be achieved relatively easily in the mixed dentition with either a removable or sectional fixed appliance (Figures 1 and 2). It will take slightly less time with the fixed appliance but both are equally effective and associated with similar stability as the dentition develops.^4^^,^^5^ Early correction of an anterior crossbite should therefore be considered in the presence of any local occlusal dysfunction and most young children will cope easily with the required treatment.

## Early correction of a Class III skeletal discrepancy

In many parts of Europe and East Asia, where Class III malocclusion is more prevalent and often has a significant maxillary skeletal component,^6^ early orthopaedic treatment is routinely undertaken. In contrast, in the UK, most of these cases are monitored and treated comprehensively in early or late adolescence, depending upon the diagnosed need for orthodontic camouflage or orthodontics combined with orthognathic surgery. While there are numerous studies showing the effects of early Class III treatment, particularly with the use of protraction headgear,^7^^,^^8^^,^^9^ many are considered to be at high risk of bias and very few have analysed patient outcomes over the long term.^10^ However, a long-term multicentre randomised clinical trial carried out in the UK has provided some interesting data relating to outcomes for Class III patients undergoing early treatment.^11^^,^^12^^,^^13^

**Fig. 3:**
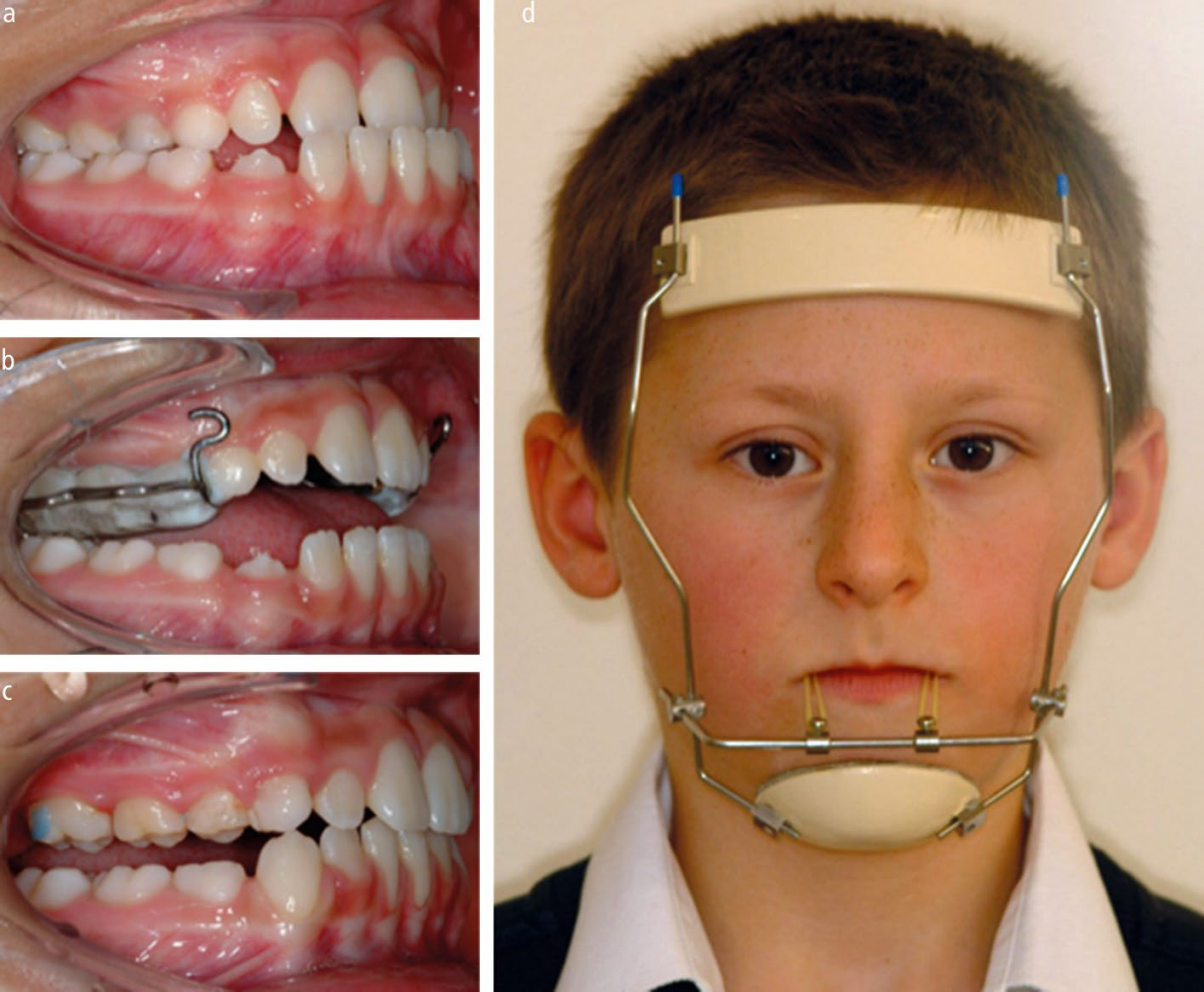
a, b, c, d) Early management of a Class III malocclusion associated with maxillary retrognathia using bonded RME and protraction headgear

This prospective study followed two groups of patients from childhood to late adolescence over a six-year period and attempted to answer the simple question of whether early intervention with protraction headgear reduces the need for orthognathic surgery in patients with a Class III malocclusion. Patients aged between 8-10 years with a skeletal Class III malocclusion and maxillary hypoplasia were randomly allocated into either a control group, where no active intervention was carried out and is standard practise in the UK, or a treatment group, that received rapid maxillary expansion (RME) combined with protraction headgear (Fig. 3). This is the largest reported trial on early Class III correction but at least three other trials comparing maxillary protraction to no treatment (observation) exist, two originating from Turkey^7^^,^^8^ and one from China.^9^

**Fig. 4:**
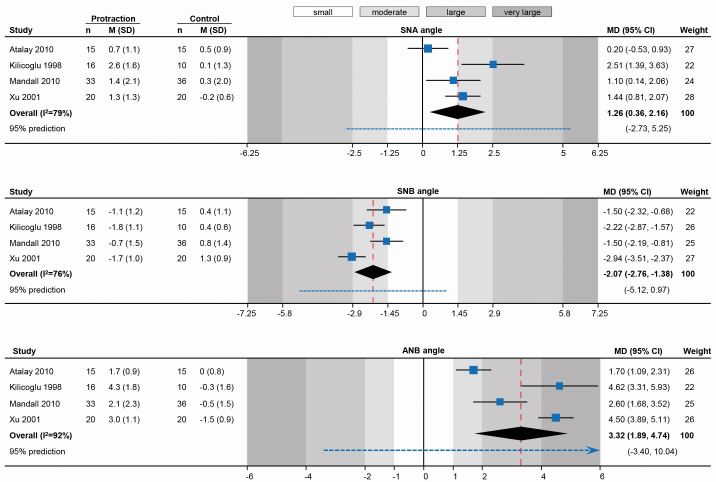
Random-effects meta-analyses of randomised trials on the short-term sagittal skeletal effects of early Class III treatment with maxillary protraction compared to no-treatment control (observation). (CI: confidence interval; M: mean; MD: mean difference; SD: standard deviation)

**Fig. 5:**
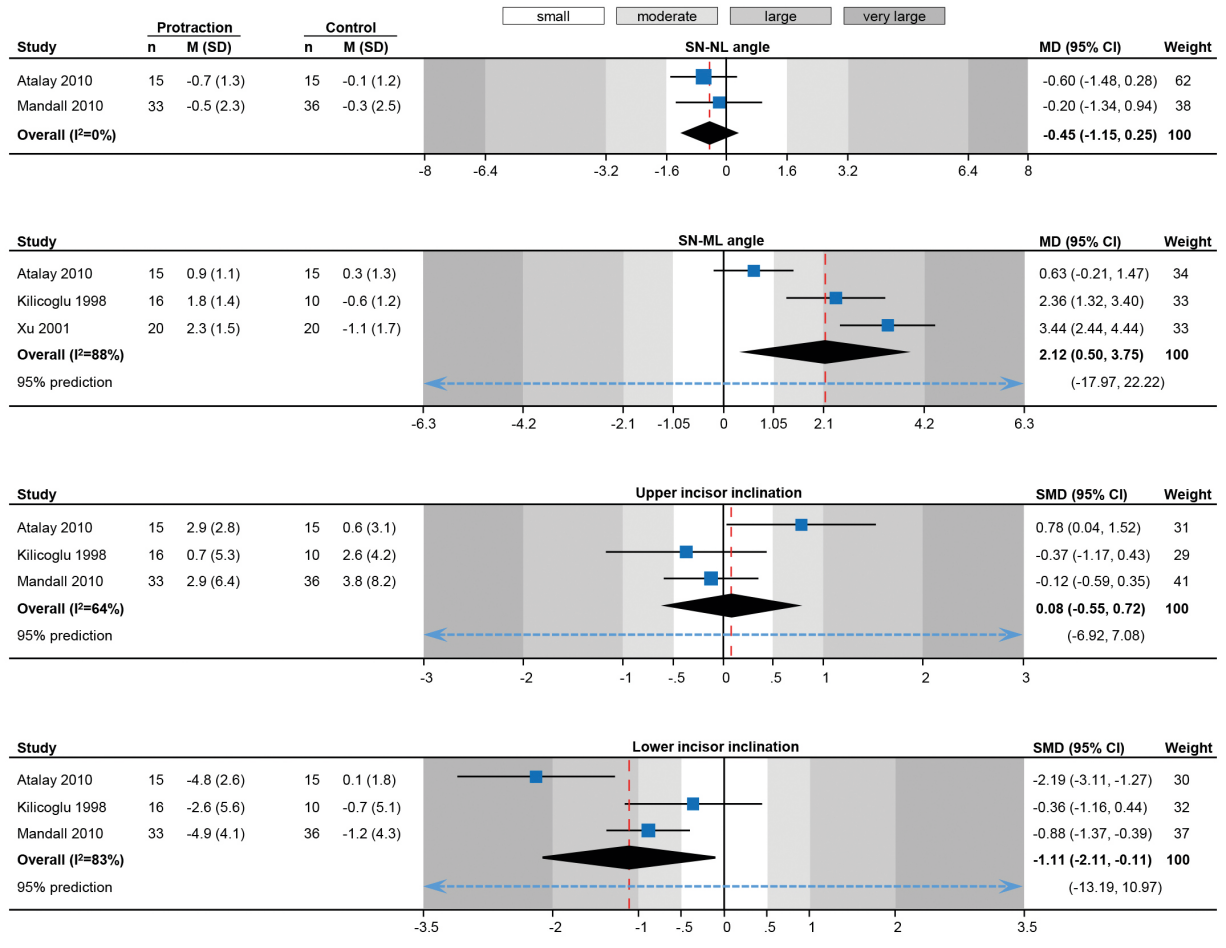
Random-effects meta-analyses of randomised trials on the short term vertical skeletal and dental effects of early Class III treatment with maxillary protraction compared to no-treatment control (observation). (CI: confidence interval; M: mean; MD: mean difference; SD: standard deviation; SMD: standardised mean difference)

Summarising evidence on the short-term effects of maxillary protraction from these existing trials (Table 1) hints at the *modus operandi* of this early correction. It seems that maxillary protraction leads to a small-to-moderate improvement in the sagittal position of the maxilla (mean difference [MD]: +1.3º in SNA angle) and a moderate improvement in the position of the mandible (MD: -2.1º in SNB angle), which leads to a total moderate to large improvement in the jaw relationship (MD: +3.3º in ANB angle) (Fig. 4). Ideally, purely orthopaedic correction of the sagittal discrepancy is desirable, without any undesired effects in terms of dental compensation or loss of vertical control. It seems that maxillary protraction does exert a minor, non-significant influence on maxillary inclination and slight opening of the mandibular plane angle (MD: +2.1º in SN-ML angle) (Fig. 5). Although inclination of the upper incisors does not seem to be significantly influenced, the lower incisors are retroclined through treatment (MD: -4.8º in L1-ML angle; back-translated from a standardised mean difference of -1.11). It seems therefore, that this early correction is achieved partly through improvement in jaw position and partly through dentoalveolar compensation of the lower incisors. Interestingly, meta-regression according to the duration of active traction indicates a statistically significant dose-response relationship where ANB angle increases by 0.87º for each additional month of traction (95% confidence interval [CI]: -0.24-1.98º; P = 0.08).

**Table 1:** Random-effects meta-analyses (restricted maximum likelihood method) of randomised trials on short-term (post-treatment) effects of early Class III treatment with maxillary protraction compared to no-treatment control (observation); data openly available^16^

**Landmark**	**Trials (n) (patients)**	**Effect**	**95% CI**	**P**	**I2** **(95% CI)**	**t2** **(95% CI)**	**95% prediction**
SNA angle	n = 4 (165)	MD = 1.26	0.36, 2.16	0.006	79% (18%, 98%)	0.65 (0.04, 7.42)	-2.73, 5.52
SNB angle	n = 4 (165)	MD = -2.07	-2.76, -1.38	<0.001	76% (20%, 97%)	0.38 (0.03, 4.14)	-5.12, 0.97
ANB angle	n = 4 (165)	MD = 3.32	1.89, 4.74	<0.001	92% (73%, 99%)	1.91 (0.42, 18.08)	-3.40, 10.04
SN-NL angle	n = 2 (99)	MD = 0.45	-1.15, 0.25	0.21	0% (0%, 98%)	0 (0, 16.54)	-
SN-ML angle	n = 3 (96)	MD = 2.12	0.50, 3.75	0.01	88% (51%, 99%)	1.82 (0.24, 35.42)	-17.97, 22.22
Upper incisor inclination	n = 3 (125)	SMD^*^ = 0.08	-0.55, 0.72	0.80	64% (0%, 98%)	0.20 (0, 5.86)	-6.92, 7.08
Lower incisor inclination	n = 3 (125)	SMD^*^ = 1.11	-2.11, -0.11	0.03	83% (0%, 99%)	0.64 (0, 14.53)	-13.19, 10.97
Key: CI = Confidence interval MD = Mean difference SMD = Standardised mean difference * = Standardised effect used to pool together different angles measuring similar outcomes (U1-NA & U1-NL or L1-NB & L1-ML)

The patients within the Mandall trial^11^ were also reviewed at three years^13^ and then six years,^12^ by which time they had reached late adolescence and were past their pubertal growth spurt. The majority of patients in the protraction group had their reverse overjet successfully corrected and at the three-year review, had identifiable morphological cephalometric differences with an increased ANB (MD: +1.4°; 95% CI: 0.4-2.4°; P = 0.004) and a downwards and backwards rotation of the maxilla identified through superimposition (MD: +8.4°; 95% CI: 6.7-10.2°; P <0.001). However, at six-year review, apart from the rotational effects (+8.2° for the maxilla; +6.7° for the mandible), these differences had all but disappeared when compared to the control group - some of who had, by then, undergone conventional orthodontic treatment.^12^ Interestingly, 68% of the protraction headgear group had a positive overjet compared to half of the control group. It is also interesting to note that an older trial had indicated that modifying the direction of applied force so that it passes through the maxillary centre of resistance might be beneficial compared to conventional intraoral force application (30° forwards/downwards) in terms of skeletal correction (+1.3° in ANB), maxillary rotation (-2.7° in SN-NL) and incisor protrusion (-10.1° in SN-U1).^14^

The original research question of the Mandall trial was whether the use of protraction headgear in Class III patients during childhood ultimately reduces the need for orthognathic surgery.^11^ As there were no defined cephalometric criteria that could absolutely determine this, an assessment was made by a group of experienced consultant orthodontists who routinely treat patients requiring orthognathic surgery. They were provided with complete records for the patients and were blinded to which group they belonged to, being asked simply to assess whether in their opinion, surgery was required to comprehensively treat the patient to the best facial and occlusal result. On this basis, 64% of the control group compared to 36% of the protraction headgear group were regarded as requiring surgery.^12^ This should be considered a moderate to large benefit (relative risk: 0.55) of early intervention in the management of selected Class III cases.

## Predicting the success of early intervention in the management of Class III patients

Is it possible to reliably identify those Class III patients who may benefit from early treatment in the long term? Numerous cephalometric studies comparing outcomes at the end of adolescent growth with the original presenting malocclusion have attempted to create algorithms that may make this prediction in childhood possible. However, significant individual variation in facial growth patterns mean that this is difficult, especially for those cases where early treatment is ultimately unsuccessful.^15^ However, it does appear that high angle cases with increased lower anterior face height and reduced overbite at the start of treatment are more likely to respond poorly to early treatment and ultimately outgrow the positive effects of it.

So what information does this provide us with to help inform clinical decision-making? Existing studies have certainly shown that protraction headgear is effective over the short term, but longer term, many of the initial morphological effects disappear with growth. However, the use of combined RME and early protraction headgear does seem to reduce the perceived need for orthognathic surgery in this group of patients by around a half. This information can be shared with patients and their parents, allowing them to make an informed choice when deciding whether to undertake early treatment. Indeed, the wearing of reverse headgear for the required amount of time to institute correction does require significant commitment from the patient.

## Conclusions

This short review has focused on the early management of Class III malocclusion. For Pseudo-Class III malocclusion associated with a local crossbite, early treatment in the mixed dentition can be effective. For those Class III discrepancies associated with maxillary retrusion, early treatment with protraction headgear can reduce the need for orthognathic surgery in the late teenage years. However, the presence of increased vertical proportions and a reduced overbite are indicators that early intervention might be less successful over the longer term.
